# Auricular Acupressure to Improve Menstrual Pain and Menstrual Distress and Heart Rate Variability for Primary Dysmenorrhea in Youth with Stress

**DOI:** 10.1155/2013/138537

**Published:** 2013-12-12

**Authors:** Yu-Jen Wang, Chin-Che Hsu, Mei-Ling Yeh, Jaung-Geng Lin

**Affiliations:** ^1^Department of Nursing, Chang Gung University of Sciences and Technology, Taoyuan, Taiwan; ^2^School of Nursing, National Taipei University of Nursing and Health Sciences, B402, No. 365 Minde Road, Taipei 11219, Taiwan; ^3^Department of Dermatology, Kaohsiung Medical College Hospital, Kaohsiung, Taiwan; ^4^School of Chinese Medicine-Acupuncture Science, China Medical University, 91 Hsueh-Shih Road, Taichung 40402, Taiwan

## Abstract

*Background*. Dysmenorrhea and accompanying symptoms can have a negative impact on academic achievement, physical activity and functioning, and quality of life. Unfortunately, stress increases the sensitivity and severity of pain, activating sympathetic responses while inhibiting parasympathetic responses. *Objective*. This study used objective, physiological measurements to evaluate the effects of auricular acupressure on menstrual pain and menstrual distress in young college students with primary dysmenorrhea across two menstrual cycles. The aim was to determine if significant differences could be detected between the intervention and follow-up phases after controlling life stress. *Design*. A one-group experimental research design was used, and repeated measurements and followups were done. Thirty-two women completed questionnaires and physiological parameters were measured. *Results*. Significant differences between the intervention and follow-up phases were found for high frequency (HF) and blood pressure on day 1 and no significant differences in menstrual pain and menstrual distress, heart rate variability, low frequency (LF), LF/HF ratio, or heart rate. *Conclusion*. Auricular acupressure effectively increases parasympathetic activity to maintain autonomic function homeostasis in young women with primary dysmenorrhea and may have a value in alleviating menstrual pain and menstrual distress in a high-stress life. Future studies should consider stress, stimulus dose of auricular acupressure, severity of menstrual pain, and a longitudinal research design.

## 1. Introduction

The prevalence of dysmenorrhea in adolescents and young women ranges between 40% and 90% and varies with age, country of residence, and population density [1–5]. Dysmenorrhea refers to cramp-like, dull, and throbbing pain that emanates from the lower abdomen [6, 7], often accompanied by nausea, vomiting, headaches, backaches, weakness, diarrhea, sleeplessness, or nervousness [8]. Such debilitating symptoms limit daily activity [9] leading to short-term school absenteeism and have negative consequences on health-related quality of life [9, 10]. In addition, dysmenorrhea is associated with decreased exercise performance [11], increased pain sensitivity [12], and changes in gray-matter volume [13] and brain metabolism [14]. Alleviating menstrual pain and menstrual distress is a critical women's health concern. Dysmenorrhea is significantly and positively associated to perceived levels of stress, occurring with an odds ratio of 2.4 in high-stress female populations compared to low-stress populations [15]. Stress refers to a state of threatened homeostasis and ubiquitous presence [16]. It tends to increase a person's sensitivity to pain although there is wide variability among individuals [17]. Dysmenorrhea has been found by many to be associated with psychological stress [18, 19], as well as stress from daily life [20] and work [21, 22].

Stress and pain both activate the sympathetic nervous system to release epinephrine and norepinephrine, which increase heart rate (HR), cardiac contractility, vascular smooth muscle contraction, and blood pressure (BP) [16, 23]. At the same time, stress and pain reduce activation of the parasympathetic nervous system (PNS) responses [24, 25]. Heart rate variability (HRV) is a convenient noninvasive method for measuring overall autonomic nervous system activity, based on interactions between the sympathetic and parasympathetic nervous systems [26]. HRV measurements incorporate a low-frequency (LF) component that represents sympathetic nerve activity, and a high-frequency (HF) component reflecting vagal activity. The LF/HF ratio mirrors the sympathovagal balance and reflects modulations in sympathetic activity [27]. HR and BP must also be considered as elements of the sympathetic reaction [16]. Autonomic fluctuations in response to pain usually lead to changes in cardiovascular parameters, such as increases in BP [28, 29] and HR [24, 30] and a decrease in HRV [24, 30]. Women experiencing primary dysmenorrhea possess low parasympathetic nerve activity throughout their menstrual cycle [31], but such activity increases after acupuncture stimulation [32]. In healthy women, however, there is no association between pain perception and HR [33]. Investigating the relationship between pain and autonomic regulation in women with dysmenorrhea is the focus of this study.

Approximately 67% of young females with primary dysmenorrhea take analgesic drugs [34], which can only alleviate pain temporarily [35]. Nonsteroidal anti-inflammatory drugs (NSAIDs) and oral contraceptives pills (OCPs) are prescribed for the relief of menstrual pain, and a systematic review study concluded that such treatments are effective [7]; however, it was noted that NSAIDs and OCPs can induce or exacerbate preexisting hypertension [36]. NSAIDs are associated with a variety of adverse effects including gastrointestinal disorders, nephrotoxic, and hepatotoxic effects, hematologic abnormalities, and fluid retention [37] and fail to alleviate menstrual pain in 20% to 25% of women [38]. OCPs also have a host of side effects including nausea, vomiting, headaches, breast tenderness, acne, weight gain, and depression [39]. Therefore, the search for an alternative yet effective nonpharmacological interventions to relieve pain in dysmenorrhea is necessary. The result of a recent systematic review and meta-analysis indicates that acupoint stimulation is an effective intervention for primary dysmenorrhea [40]. Auricular acupressure is a noninvasive acupoint stimulation that transmits signals to the brain and to specific organs to modulate and harmonize physiological function [41]. The positive effects of auricular acupressure to alleviate menstrual pain and menstrual distress have been studied and reported [42–46], although some of these studies lacked methodological rigor [40] or failed to measure physiological indicators [45].

## 2. Purpose Statements 

In this study, physiological parameters were measured across two menstrual cycles in young college students with primary dysmenorrhea in order to objectively evaluate the effects of auricular acupressure. We hypothesized that after controlling for life stress, significant differences would be identified for menstrual pain, menstrual distress, and HRV physiological parameters between the intervention and follow-up periods.

## 3. Materials and Methods

### 3.1. Research Design and Participants

**Figure 1:**
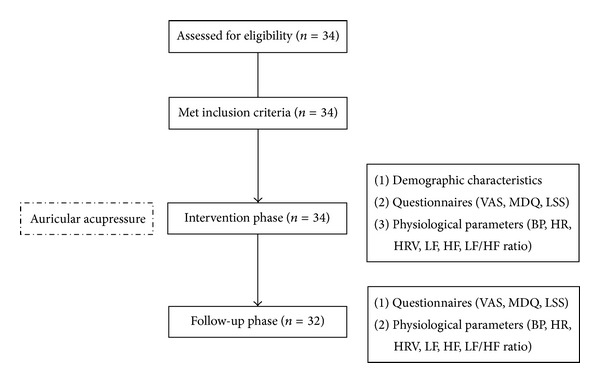
Study flowchart.

A single-group experimental research design was used, and repeated measures were done in the intervention and follow-up phases. A convenient sample of young college students with primary dysmenorrhea was recruited from a college in northern Taiwan. All participants were given auricular acupressure to relieve their menstrual pain and menstrual distress for a certain period. The inclusion criteria were (1) 18 to 25 years of age, (2) menstrual-cycle duration between 25 and 40 days, (3) body mass index between 18.5 and 24.9 kg/m^2^, and (4) pain score 3. Women meeting the following criteria were excluded: (1) diagnosis of pelvic disease, gynecological disease or surgery, or secondary dysmenorrhea, (2) chronic disease, such as diabetes, renal disease, or cardiovascular disease, (3) serious arrhythmia or pacemaker user, (4) habitual smoking or consumption of stimulant beverages such as tea, coffee, or alcohol, and (5) swelling, infections, and ulcers in both ears. Sample size was estimated using G Power software and was based on the study of Yeh et al. [45], which indicated that auricular acupressure was effective in relieving menstrual pain, providing an average (±standard deviation) improvement of 5.14 ± 2.32 points in the visual analog pain score. An estimated sample size of 32 would be required to demonstrate significant at the 5% probability level with 80% power.  Figure 1 shows the flowchart of research design and participants of this study. Menstrual pain, menstrual distress, and HRV physiological parameters were measured.

### 3.2. Intervention

Based on a literature review of auricular acupressure for treating dysmenorrhea, six common auricular acupoints were used: *internal genitals*,* endocrine*,* shenmen*,* sympathesis*,* liver*, and* kidney*. The *internal genitals* acupoint was selected to dredge the meridian and normalize circulation, eliminate stasis, and alleviate pain. The *endocrine* acupoint was targeted to harmonize physical function, regulate menstruation, and improve menstrual distress. The *shenmen* acupoint was used to reduce pain and provide tranquility [47], while the *sympathesis* acupoint was to normalize autonomic nervous system and vasomotor functions, relieve muscle spasm, and enhance the analgesic effect [47, 48]. The *liver* acupoint was stimulated to disperse stagnated liver qi for relieving stagnation and to regulate the flow of qi for alleviating pain, and the *kidney* acupoint was stimulated to coordinate Chong and conception vessels, invigorate kidney qi, and active qi and blood for the relief of pain [48]. Cowherb seeds with adhesive patches were embedded on the specific acupoints two to three days before menstruation, and the application of pressure was initiated at the onset of menstrual pain. All participants were instructed to press each acupoint for 1 minute, 4 times per day until they achieved relief of menstrual pain. They were also informed that they may experience various sensations while applying pressure: numbness, swelling, mild pain, or warmth. The adhesive patch and Cowherb seed were removed accordingly only if pain had been relieved for 48 hours.

### 3.3. Measures

Menstrual pain was evaluated using a 100 mm horizontal visual analogue scale (VAS) where 0 represented no pain and 100 indicated unbearable pain. Participants were instructed to indicate a point on the scale corresponding to the pain intensity. The distance from the left end to the selected point was measured to calculate the pain score in millimeters. Higher scores represent higher intensity of menstrual pain. Menstrual distress was measured using the modified 16-item Menstrual Distress Questionnaire (MDQ) that assesses menstruation related symptoms (pain, water retention, and autonomic reactions) during the premenstrual and menstrual periods [49]. Each item was scored from 1 (no symptoms) to 4 (severe symptoms), with higher scores reflecting higher severity of distress. Cronbach's alpha for the internal consistency reliability was 0.83 in the earlier study [45] and 0.80 in this study.

HRV was measured using an ANSWatch wrist monitor (Taiwan Scientific Co., Taipei, Taiwan). This monitor uses multiple piezoelectrical sensors in the cuff to measure blood pressure waveforms in the radial artery. HRV, LF, HF, and LF/HF ratio were analyzed based on the international standard [27]. The accuracy of ANS monitor was represented by the correlation between HRV parameters and EKG [50]. HRV measurements were taken between the hours of 8 pm and 10 pm, and participants were instructed to refrain from eating, drinking stimulant beverages (such as tea, coffee, and alcohol), smoking cigarettes, and exercising 2 hours prior to the measurements. Participants were first subjected to rest quietly for 10 minutes in a sitting posture; and were then assisted to wear the ANS monitor on the left wrist, instructed to close eyes, to relax and remain quiet, and to not move for 7 minutes while waveforms were being recorded. HR and BP were measured at the same time. Data were downloaded to a notebook using the ANS Watch Manager Pro software.

The Chinese version of the Life Stress Scale (LSS) [51] was used to measure life stress over the preceding month. The LSS consisted of 29 items categorized into six subscales: including academic stress, family stress, interpersonal stress, emotional stress, employment stress, and self-cognition stress. Each item was scored from 0 (no stress) to 4 (extremely stressful). Higher scores indicated a higher level of life stress. Cronbach's alpha was 0.92 from a previous study [51] and 0.92 in this study.

### 3.4. Procedures and Data Analysis

The study protocol and design were reviewed and approved by the Chang Gung Medical Foundation Institutional Review Board (reference number: 100-2728A3). Verbal and written informed consent were obtained from all participants after informing them of the study design, intervention, data collection, and the rights of the participants. They were made aware that all data remained confidential at all times and that they were free to withdraw at any time during the study without affecting their academic grades. Interventions and data collection were performed by the researcher and trained research assistants. Menstrual pain and HRV parameters were measured repeatedly during the intervention phase and the follow-up phase; while menstrual distress and life stress levels were measured once at the end of menstruation during the intervention phase and follow-up phase. Day 1 indicated the day of greatest menstrual pain during the menstrual cycle. Adverse effects of the intervention were also recorded. Data were analyzed using IBM SPSS 20.0 for Windows. Descriptive statistics was used to analyze demographics. A paired *t*-test was used to test for differences between the two phases in VAS, MDQ, physiological parameters, and LSS. *P* < 0.05 was considered statistically significant.

## 4. Results

Thirty-four women were recruited for the study, two of whom later withdrew due to personal reasons. Thus, 32 women completed the study, and the attrition rate was 5.88%.  Table 1 shows the demographic characteristics of the participants. The mean age for women in the study was 20.78 ± 1.53 years, and the mean age at menarche was 11.94 ± 0.91 years. The average menstrual cycle length was 30.97 ± 3.28 days, and the mean menses duration was 6.28 ± 1.37 days. Most participants had regular menstruation and first experienced menstrual pain less than two years after menarche. Menstrual pain occurred in the first two days of menses. Past pain intensity was 7.75 ± 1.53.

**Figure 2:**
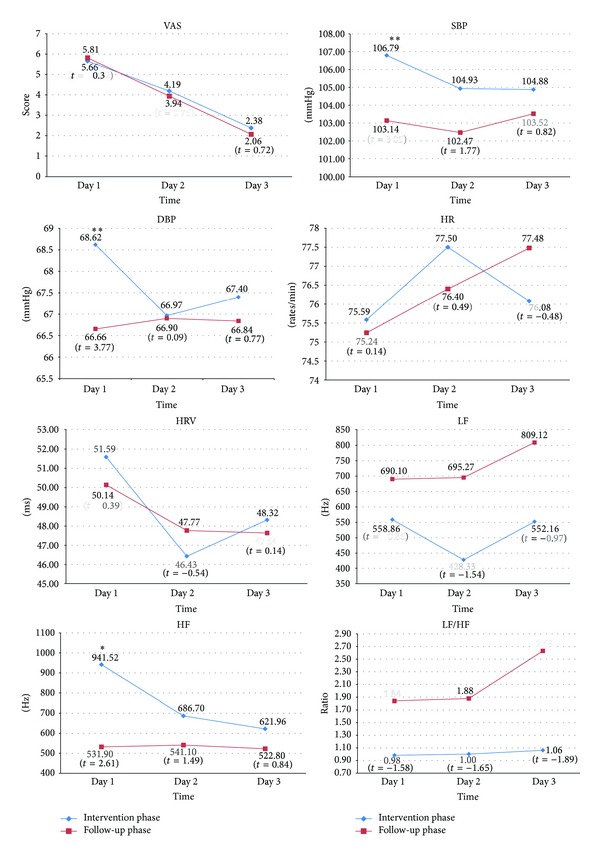
The comparison of VAS and physiological parameters on days 1–3, *P < 0.05, **P < 0.01.

Table 2 shows the comparison of MDQ and LSS between the intervention phase and follow-up phase. The MDQ was slightly higher in the intervention phase, but the difference was not statistically significant (*P* = 0.26). LSS was found to be significantly higher during the intervention phase compared to the follow-up phase (*P* = 0.001).  Figure 2 shows the comparison of VAS and physiological parameters on days 1–3 of the two phases. Significant differences between the two phases were found on day 1 for HF (*P* = 0.01), systolic BP (*P* = 0.005), and diastolic BP (*P* = 0.001), but not for menstrual pain (*P* = 0.75), HRV (*P* = 0.70), LF (*P* = 0.40), LF/HF ratio (*P* = 0.12), and HR (*P* = 0.89). No significant differences were found for VAS or other parameters on days 2 and 3 (*P* > 0.05).

## 5. Discussion

The average age of menarche for the participants in this study was 12 years. Most participants first experienced menstrual pain within two years of menarche, and the menstrual pain continued for nearly 7 to 8 years. Menstrual pain of the participants in this study persisted for 2 to 3 days during the menstrual cycle, which is consistent with other studies [37, 52]. Menstrual pain was similar during the first three days of the cycle in both the intervention phase and follow-up phase, whether auricular acupressure was used or not. For both the intervention and follow-up phases, menstrual pain level was at 5.74, 4.07, and 2.22 on days 1, 2, and 3, respectively. Without considering stress, this finding is not consistent with the effects of auricular acupressure for improving dysmenorrhea reported in other studies [42, 45, 46]. Indeed, life stress impacts menstrual pain in the intervention phase. With life stress influences [17], menstrual pain would be more serious if auricular acupressure was not given. Therefore, the effect of auricular acupressure on reducing menstrual pain was insignificant. Comparing to the results from follow-up phase with low life stress, this study supports that the effect of auricular acupressure is reduction of menstrual pain.

In addition, participants have an obviously lowered pain perception in the intervention (5.66) phase and follow-up (5.81) phase compared to the average pain intensity (7.75) from the previous month prior to intervention. This indicates that auricular acupressure may improve menstrual pain during the intervention phase and that the effect may persist through the follow-up phase. One the other hand, the effects of auricular stimulation for acute pain were immediate rather than long term while comparing standard medical care alone or in combination with auricular acupuncture [53]. It should be noted that no obvious reduction of menstrual pain was found between the intervention and follow-up phases in this study, and it seems that the stimulant dose of auricular acupressure is insufficient to alleviate menstrual pain. This study suggests that the protocol for acupressure application can be considered multiple times per day that may improve the overall duration of the effect. Further studies should also increase stimulus dose, including acupressure frequency, duration, and intensity and examine a prolonged course of intervention for achieving a long-term effect.

This study found that menstrual distress from menstrual-related symptoms was due primarily to pain, water retention, and autonomic reactions. Although auricular acupressure was provided, menstrual distress during the intervention phase was similar to the follow-up phase; making the result inconsistent with previous studies [42, 45, 46]. As mentioned previously, the participants would have had more pain and autonomic responses during the high life stress of the intervention phase, which should also be observed as a factor affecting the results of menstrual distress. Thus, there were no obvious effects of auricular acupressure in reducing menstrual distress. It is not surprising this is inconsistent with other studies that found that acupoint stimulation decreased water retention and the autonomic reactions of menstrual distress [53, 54].

This study supports the effect of auricular acupressure to effectively maintain the autonomic homeostasis in young women with primary dysmenorrhea, in terms of increasing the HF activity that regulates the menstrual cycle, but not LH activity or the LF/HF ratio. This is in agreement with other studies in postmenopausal women with insomnia [55] and in healthy adults [56, 57]. Auricular acupressure may stimulate the auricular branch of the vagus nerve, leading to an increase in parasympathetic activity and modifying both autonomic and central nervous system activity [58]. HF reflects vagal activity that contributes to the maintenance of homeostasis during menstrual pain. This finding is similar to that of other studies in which HRV was increased and LF/HF ratio was unchanged in healthy adults [57] and individuals with chronic insomnia [59], while sympathetic activity remained unchanged in healthy adults [56]. In contrast, LF signals reflect sympathetic activity. Other studies have found an increase in LF due to an increase in the intensity of the BP regulatory mechanism [60] or a decrease in LF due to response for improving insomnia [55]. In comparison to the reference LF/HF ratio of 0.8 to 1.5, we measured ratios of 0.98 to 1.06 in the intervention phase and 1.84 to 2.63 in the follow-up phase, which indicate a balance of sympathetic and vagal activity during the intervention phase. Therefore, this finding indicates that auricular acupressure increases parasympathetic activity and regulates the homeostasis of autonomic function in young women with primary dysmenorrhea.

Systolic and diastolic BP, but not HR, was significantly elevated on day 1 between two phases. This is inconsistent with previous studies that reported either a decrease [57] or no change in BP [61, 62]. Many studies have shown a decrease in HR for healthy adults [57, 60], individuals with chronic insomnia [59], and those experiencing anxiety associated with dental extractions [63]. Others have reported no change in HR for healthy adults [56, 61] and those experiencing anxiety before surgery [62]. Activating the vagus nerve typically causes a reduction in HR and BP [58]. However, in the presence of pain and stress, HR and BP are increased in response to vagus nerve stimulation [16, 23]. Accordingly, participants with high stress and pain in the intervention phase could activate sympathetic reactions, leading to an elevation of BP, while HR would remain unchanged due to acupoint-stimulation related simultaneous activation of the parasympathetic nervous system. Various ages, physical conditions, auricular stimulus doses, and measure time points may lead to different results. In addition, previous studies have shown no relationship between HR and pain perception in women [33]. Thus, HR may not be an appropriate indicator of pain outcome for women [62].

### 5.1. Limitations

This study has some limitations. The absence of a control group and baseline measurements makes it difficult to directly relate the outcome measures to the interventions. In addition, a small sample population was from a single college, which limited our ability to perform subgroup analysis (such as mild, moderate, and severe pain), and the extent to which the results can be generalized. Because the intervention was given during one menstrual period and the outcomes were measured over two cycles only, the long-term effects are unknown.

## 6. Conclusion

Auricular acupressure is an effective noninvasive intervention that increases HF to maintain autonomic function homeostasis in young women with primary dysmenorrhea. It may be valuable in alleviating menstrual pain and menstrual distress in high-life stress conditions. Life stress can increase menstrual pain and impact the effect of auricular acupressure. Increasing the simulation requirement of auricular acupressure in high life stress conditions should be considered. Further studies considering stress, using a longitudinal randomized-controlled design, expanding recruiting sites, enlarging sample sizes, involving individuals with differences in the severity of dysmenorrhea, modifying intervention doses, and increasing additional endpoints of timing should be considered.

## Figures and Tables

**Table 1:** Participants' demographic characteristics.

Characteristics	Mean ± SD	*n* (%)
Age (years)	20.78 ± 1.53	
Age at menarche (years)	11.94 ± 0.91	
Menstrual cycle (days)	30.97 ± 3.28	
Menses duration (days)	6.28 ± 1.37	
Past pain intensity	7.75 ± 1.53	
Menstrual regularity		
Yes		25 (78.1%)
No		7 (21.9%)
Initial onset of menstrual pain		
Menarche		3 (9.4%)
<1 year after menarche		14 (43.8%)
1-2 years after menarche		9 (28.1%)
Others		6 (18.8%)
Time of dysmenorrhea		
Day before menses		8 (25%)
First 2 days in menses		23 (71.9%)
Others		1 (3.1%)

**Table 2:** Menstrual distress and life stress in the intervention and follow-up phases.

Variables	Intervention	Follow-up	Paired *t*-test
	Mean ± SD	Mean ± SD	
Menstrual distress	29.47 ± 5.95	28.31 ± 5.95	1.15
Life stress	31.97 ± 16.32	28.53 ± 16.38	3.75**

**P < 0.01.

## References

[1] Cakir M, Mungan I, Karakas T, Girisken I, Okten A (2007). Menstrual pattern and common menstrual disorders among university students in Turkey. *Pediatrics International*.

[2] Chiou M-H, Wang H-H (2008). Predictors of dysmenorrhea and self-care behavior among vocational nursing school female students. *The Journal of Nursing Research*.

[3] Dambhare DG, Wagh SV, Dudhe JY (2012). Age at menarche and menstrual cycle pattern among school adolescent girls in central India. *Global Journal of Health Science*.

[4] Houston AM, Abraham A, Huang Z, D’Angelo LJ (2006). Knowledge, attitudes, and consequences of menstrual health in urban adolescent females. *Journal of Pediatric and Adolescent Gynecology*.

[5] Okusanya BO, Garba KK, Okome GB, Ohiosimuan O (2009). Menstrual pain and associated factors amongst undergraduates of Ambrose Alli University Ekpoma, Edo State, Nigeria. *Nigerian Journal of Medicine*.

[6] Grandi G, Ferrari S, Xholli A (2012). Prevalence of menstrual pain in young women: what is dysmenorrhea. *Journal of Pain Research*.

[7] Zahradnik H-P, Hanjalic-Beck A, Groth K (2010). Nonsteroidal anti-inflammatory drugs and hormonal contraceptives for pain relief from dysmenorrhea: a review. *Contraception*.

[8] Harel Z (2008). Dysmenorrhea in adolescents. *Annals of the New York Academy of Sciences*.

[9] Ortiz MI (2010). Primary dysmenorrhea among Mexican university students: prevalence, impact and treatment. *European Journal of Obstetrics Gynecology & Reproductive Biology*.

[10] Unsal A, Ayranci U, Tozun M, Arslan G, Calik E (2010). Prevalence of dysmenorrhea and its effect on quality of life among a group of female university students. *Upsala Journal of Medical Sciences*.

[11] Chantler I, Mitchell D, Fuller A (2009). Diclofenac potassium attenuates dysmenorrhea and restores exercise performance in women with primary dysmenorrhea. *The Journal of Pain*.

[12] Iacovides S, Baker FC, Avidon I, Bentley A (2013). Women with dysmenorrhea are hypersensitivity to experimental deep muscle pain across the menstrual cycle. *The Journal of Pain*.

[13] Tu C-H, Niddam DM, Chao H-T (2010). Brain morphological changes associated with cyclic menstrual pain. *Pain*.

[14] Tu C-H, Niddam DM, Chao H-T (2009). Abnormal cerebral metabolism during menstrual pain in primary dysmenorrhea. *NeuroImage*.

[15] Wang L, Wang X, Wang W (2004). Stress and dysmenorrhoea: a population based prospective study. *Occupational and Environmental Medicine*.

[16] Page GG, Lindsey AM, Carrieri-kohlman V, Lindsey AM, West CM (2003). Stress response. *Pathophysiological Phenomena in Nursing*.

[17] Reinhardt T, Kleindienst N, Treede RD, Bohus M, Schmahl C (2013). Individual modulation of pain sensitivity under stress. *Pain Medicine*.

[18] Tavallaee M, Joffres MR, Corber SJ, Bayanzadeh M, Rad MM (2011). The prevalence of menstrual pain and associated risk factors among Iranian women. *Journal of Obstetrics and Gynaecology Research*.

[19] Yamamoto K, Okazaki A, Sakamoto Y, Funatsu M (2009). The relationship between premenstrual symptoms, menstrual pain, irregular menstrual cycles, and psychosocial stress among Japanese college students. *Journal of Physiological Anthropology*.

[20] Gordley LB, Lemasters G, Simpson SR, Yiin JH (2000). Menstrual disorder and occupational, stress, and racial factors among military personnel. *Journal of Occupational and Environmental Medicine*.

[21] László KD, Gyorffy Z, Ádám S, Csoboth C, Kopp MS (2008). Work-related stress factors and menstrual pain: a nation-wide representative survey. *Journal of Psychosomatic Obstetrics & Gynecology*.

[22] Nohara M, Momoeda M, Kubota T, Nakabayashi M (2011). Menstrual cycle and menstrual pain problems and related risk factors among Japanese female workers. *Industrial Health*.

[23] Puntillo KA, Miaskowski C, Summer G, Carrieri-kohlman V, Lindsey AM, West CM (2003). Pain. *Pathophysiological Phenomena in Nursing*.

[24] Hallman DM, Lyskov E (2012). Autonomic regulation, physical activity and perceived stress in subjects with musculoskeletal pain: 24-hour ambulatory monitoring. *International Journal of Psychophysiology*.

[25] Matthews S, Jelinek H, Vafaeiafraz S, McLachlan CS (2012). Heart rate stability and decreased parasympathetic heart rate variability in healthy young adults during perceived stress. *International Journal of Cardiology*.

[26] Zygmunt A, Stanczyk J (2010). Methods of evaluation of autonomic nervous system function. *Archives of Medical Science*.

[27] Malik M, Camm AJ, Bigger JT (1996). Heart rate variability: standards of measurement, physiological interpretation, and clinical use. Task Force of the European Society of Cardiology and the North American Society of Pacing and Electrophysiology. *European Heart Journal*.

[28] Park M-K, Watanuki S (2005). Specific physiological responses in women with severe primary dysmenorrhea during the menstrual cycle. *Journal of Physiological Anthropology and Applied Human Science*.

[29] Yilmaz U, Liu Y-W, Berger RE, Yang CC (2007). Autonomic nervous system changes in men with chronic pelvic pain syndrome. *The Journal of Urology*.

[30] Terkelsen AJ, Mølgaard H, Hansen J, Finnerup NB, Krøner K, Jensen TS (2012). Heart rate variability in complex regional pain syndrome during rest and mental and orthostatic stress. *Anesthesiology*.

[31] Hegazi M, Nasrat H (2007). Heart rate variability (HRV) in young health females with primary dysmenorrhea. *Bulletin of Akexandria Faculty of Medicine*.

[32] Kim E, Cho J-H, Jung WS, Lee S, Pak SC (2011). Effect of acupuncture on heart rate variability in primary dysmenorrheic women. *The American Journal of Chinese Medicine*.

[33] Tousignant-Laflamme Y, Rainville P, Marchand S (2005). Establishing a link between heart rate and pain in healthy subjects: a gender effect. *The Journal of Pain*.

[34] Polat A, Celik H, Gurates B (2009). Prevalence of primary dysmenorrhea in young adult female university students. *Archives of Gynecology and Obstetrics*.

[35] Jun E-M, Chang S, Kang D-H, Kim S (2007). Effects of acupressure on dysmenorrhea and skin temperature changes in college students: a non-randomized controlled trial. *International Journal of Nursing Studies*.

[36] Faselis C, Doumas M, Papademetriou V (2011). Common secondary causes of resistant hypertension and rational for treatment. *International Journal of Hypertension*.

[37] Dawood MY (2006). Primary dysmenorrhea: advances in pathogenesis and management. *Obstetrics and Gynecology*.

[38] Proctor ML, Murphy PA (2001). Herbal and dietary therapies for primary and secondary dysmenorrhoea. *Cochrane Database of Systematic Reviews*.

[39] Wong CL, Farquhar C, Roberts H, Proctor M (2009). Oral contraceptive pill for primary dysmenorrhea. *Cochrane Database of Systematic Reviews*.

[40] Chung Y-C, Chen H-H, Yeh M-L (2012). Acupoint stimulation intervention for people with primary dysmenorrhea: systematic review and meta-analysis of randomized trials. *Complementary Therapies in Medicine*.

[41] Yeh ML, Chen HH, Lin IH (2004). *Contemporary Meridians and Acupoints in Practice*.

[42] Kim SY, Lee HY (2010). Effect of the auricular acupressure therapy on dysmenorrhea of puberty girls. *Korean of Journal Women Health Nursing*.

[43] Wan Q, Lin LC, Kuo HW, Lai DY, Liu PE (2010). Auricular acupressure for dysmenorrhea. *Integrative Nursing*.

[44] Wu R-D, Zhang H-D, Lin L-F (2007). Observation on ear point taping and pressing therapy for treatment of primary dysmenorrhea. *Chinese Acupuncture & Moxibustion*.

[45] Yeh M-L, Hung Y-L, Chen H-H, Lin J-G, Wang Y-J (2013). Auricular acupressure combined with an internet-based intervention or alone for primary dysmenorrhea: a control study. *Evidence-Based Complementary and Alternative Medicine*.

[46] Yeh ML, Hung YL, Chen HH, Wang YJ (2013). Auricular acupressure for pain relief in adolescents with dysmenorrhea: a placebo-controlled study. *The Journal of Alternative and Complementary Medicine*.

[47] Fan FY, Shan CH (2009). The overview of acupoints use in auricular acupressure for treating dysmenorrhea. *Journal of Traditional Chinese Medical Literature*.

[48] Lee SL, Jin F (2008). 215 cases of treating dysmenorrhea by auricular acupressure. *Hebei Journal of Traditional Chinese Medicine*.

[49] Wang H (1991). *Study on the factors of menstrual physiological distress of junior and senior high school female students in Hsin-Chu City [M.S. thesis] [M.S. thesis]*.

[50] Sun DC, Guo YY, Liang YH Introduction of a new wrist patient monitor—ANS Watch and its clinical applications.

[51] Lu YL (2005). *A study on the life stress and stress coping strategy of the National Tai-Chung Teachers College (NTCTC) students [M.S. thesis]*.

[52] Lefebvre G, Pinsonneault O, Antao V (2005). Primary dysmenorrhea consensus guideline. *Journal of Obstetrics and Gynaecology Canada*.

[53] Goertz CMH, Niemtzow R, Burns SM, Fritts MJ, Crawford CC, Jonas WB (2006). Auricular acupuncture in the treatment of acute pain syndromes: a pilot study. *Military Medicine*.

[54] Wang M-C, Hsu M-C, Chien L-W, Kao C-H, Liu C-F (2009). Effects of auricular acupressure on menstrual symptoms and nitric oxide for women with primary dysmenorrhea. *Journal of Alternative and Complementary Medicine*.

[55] Kung Y-Y, Yang CCH, Chiu J-H, Kuo TBJ (2011). The relationship of subjective sleep quality and cardiac autonomic nervous system in postmenopausal women with insomnia under auricular acupressure. *Menopause*.

[56] Haker E, Egekvist H, Bjerring P (2000). Effect of sensory stimulation (acupuncture) on sympathetic and parasympathetic activities in healthy subjects. *Journal of the Autonomic Nervous System*.

[57] Hsu C-C, Weng C-S, Sun M-F, Shyu L-Y, Hu W-C, Chang Y-H (2007). Evaluation of scalp and auricular acupuncture on EEG, HRV, and PRV. *The American Journal of Chinese Medicine*.

[58] He W, Wang X, Shi H (2012). Auricular acupuncture and vagal regulation. *Evidence-Based Complementary and Alternative Medicine*.

[59] Wang L, Cheng W, Sun Z (2013). Ear acupressure, heart rate, and heart rate variability in patients with insomnia. *Evidence-Based Complementary and Alternative Medicine*.

[60] Gao X-Y, Wang L, Gaischek I, Michenthaler Y, Zhu B, Litscher G (2012). Brain-modulated effects of auricular acupressure on the regulation of autonomic function in healthy volunteers. *Evidence-Based Complementary and Alternative Medicine*.

[61] Kraus T, Hösl K, Kiess O, Schanze A, Kornhuber J, Forster C (2007). BOLD fMRI deactivation of limbic and temporal brain structures and mood enhancing effect by transcutaneous vagus nerve stimulation. *Journal of Neural Transmission*.

[62] Wang S-M, Kain ZN (2001). Auricular acupuncture: a potential treatment for anxiety. *Anesthesia and Analgesia*.

[63] Karst M, Winterhalter M, Münte S (2007). Auricular acupuncture for dental anxiety: a randomized controlled trial. *Anesthesia and Analgesia*.

